# A phase 1 trial of fuzuloparib in combination with apatinib for advanced ovarian and triple-negative breast cancer: efficacy, safety, pharmacokinetics and germline *BRCA* mutation analysis

**DOI:** 10.1186/s12916-023-03046-8

**Published:** 2023-09-29

**Authors:** Yaxin Liu, Wei Wang, Rutie Yin, Youzhong Zhang, Yu Zhang, Keqiang Zhang, Hongming Pan, Ke Wang, Ge Lou, Guiling Li, Ruyan Zhang, Kun Li, Jing Rao, Ben Zhang, Yuting Wang, Quanren Wang, Yunong Gao, Huiping Li

**Affiliations:** 1 Key Laboratory of Carcinogenesis and Translational Research (Ministry of Education), Department of Breast Oncology, Peking University Cancer Hospital and Institute, No. 52 Fucheng Road, Beijing, China; 2 Key Laboratory of Carcinogenesis and Translational Research (Ministry of Education), Department of Gynecologic Cancer Surgery Unit, Peking University Cancer Hospital and Institute, No. 52 Fucheng Road, Beijing, China; 3 Radiation Therapy and Chemotherapy for Gynecologic Cancer, West China Second University Hospital, Sichuan University, Chengdu, China; 4 Department of Obstetrics and Gynecology, Qilu Hospital of Shangdong University, Jinan, China; 5 Medical Ethics Committee, Xiangya Hospital, Central South University, Changsha, China; 6 Gynecologic Oncology Ward V, Hunan Cancer Hospital, Changsha, China; 7 Department of Medical Oncology, Sir Run Run Shaw Hospital, Zhejiang University School of Medicine, Hangzhou, China; 8 Department of Gynecologic Oncology, Tianjin Medical University Cancer Institute and Hospital, Tianjin, China; 9 Gynecology Ward 1, Harbin Medical University Cancer Hospital, Harbin, China; 10 Department of Gynecologic Oncology, Union Hospital, Tongji Medical College, Huazhong University of Science and Technology, Wuhan, China; 11 Jiangsu Hengrui Pharmaceuticals Co., Ltd, Shanghai, China

**Keywords:** Ovarian cancer, Triple-negative breast cancer, PARP inhibitor, Anti-angiogenic therapy, g*BRCA*

## Abstract

**Background:**

The effect of the combination of an anti-angiogenic agent with a poly (ADP-ribose) polymerase (PARP) inhibitor in cancer treatment is unclear. We assessed the oral combination of fuzuloparib, a PARP inhibitor, and apatinib, a VEGFR2 inhibitor for treating advanced ovarian cancer (OC) or triple-negative breast cancer (TNBC).

**Methods:**

This dose-escalation and pharmacokinetics-expansion phase 1 trial was conducted in China. We used a standard 3 + 3 dose-escalation design, with 7 dose levels tested. Patients received fuzuloparib orally twice daily, and apatinib orally once daily. The study objectives were to determine the safety profile, recommended phase 2 dose (RP2D), pharmacokinetics, preliminary efficacy, and efficacy in relation to germline *BRCA* mutation (g*BRCA*^mut^).

**Results:**

Fifty-two pre-treated patients were enrolled (30 OC/22 TNBC). 5 (9.6%) patients had complete response, 14 (26.9%) had partial response, and 15 (28.8%) had stable disease. Objective response rate (ORR) and disease control rate were 36.5% (95% CI 23.6–51.0) and 65.4% (95% CI 50.9–78.0), respectively. At the highest dose level of fuzuloparib 100 mg plus apatinib 500 mg, the ORR was 50.0% (4/8; 95% CI 15.7–84.3); this dose was determined to be the RP2D. Patients with g*BRCA*^mut^ had higher ORR and longer median progression-free survival (PFS) than those with g*BRCA*^wt^, both in OC (ORR, 62.5% [5/8] vs 40.9% [9/22]; PFS, 9.4 vs 6.7 months) and TNBC (ORR, 66.7% [2/3] vs 15.8% [3/19]; PFS, 5.6 vs 2.8 months). Two dose-limiting toxicities occurred: grade 4 febrile neutropenia (fuzuloparib 100 mg plus apatinib 250 mg) and thrombocytopenia (fuzuloparib 100 mg plus apatinib 375 mg). Maximum tolerated dose was not reached. The most common treatment-related grade ≥ 3 toxicities in all patients were hypertension (19.2%), anaemia (13.5%), and decreased platelet count (5.8%). Exposure of apatinib increased proportionally with increasing dose ranging from 250 to 500 mg, when combined with fuzuloparib 100 mg.

**Conclusions:**

Fuzuloparib plus apatinib had acceptable safety in patients with advanced OC or TNBC. Fuzuloparib 100 mg bid plus apatinib 500 mg qd was established as the RP2D. With the promising clinical activity observed, this combination is warranted to be further explored as a potential alternative to chemotherapy.

**Trial registration:**

ClinicalTrials.gov, NCT03075462 (Mar. 9, 2017).

**Supplementary Information:**

The online version contains supplementary material available at 10.1186/s12916-023-03046-8.

## Background

According to the Cancer Genome Atlas, aggressive tumors such as high-grade serous ovarian cancer (HGSOC) and triple-negative breast cancer (TNBC) exhibit genomic characteristics associated with deficiencies in the homologous recombination (HR) repair pathway (e.g. BRCA deficiency) [1, 2]. Poly (ADP-ribose) polymerase (PARP) inhibitors cause the accumulation of double-strand breaks that cannot be effectively repaired, leading to synthetic lethality in HR-deficient cells [3, 4]. PARP inhibitors are currently indicated as treatment for *BRCA*-mutated (*BRCA*^mut^) OC and HER2- BC, as well as maintenance treatment for OC after response to platinum-based chemotherapy, regardless of *BRCA*^mut^ [5–7]. Therefore, BRCA detection becomes particularly important. To further expand the scope of potential patients who could benefit from PARP inhibitors (e.g. those with platinum-resistant, non-*BRCA*^mut^ disease) and to spare the toxicities from repeated platinum therapy, there is a need for exploration of effective chemo-free combination regimens.

Anti-angiogenic therapy could induce hypoxia in the tumor microenvironment, cause genetic instability and downregulation of BRCA1/2 and lead to HR deficiency, thereby potentiating response to PARP inhibitors [8–10]. The combination of anti-angiogenic therapy and a PARP inhibitor has been evaluated in several clinical trials. In phase 2 trials of platinum-sensitive recurrent OC, the addition of the anti-vascular endothelial growth factor (VEGF) antibody bevacizumab to niraparib, or the VEGF receptor (VEGFR) inhibitor cediranib to olaparib, improved progression-free survival (PFS) [11–13]. Promising anti-tumor activity was also observed with the olaparib-cediranib combination in patients with platinum-resistant OC, regardless of *BRCA* mutation status [14–16]. However, the administration of bevacizumab requires intravenous infusion, which may decrease the convenience of treatment. In addition, although cediranib is an oral tyrosine kinase inhibitor (TKI), the olaparib-cediranib combination has been associated with tolerability issues, leading to dose reduction in over 70% of OC patients [13].

Fuzuloparib (formerly known as fluzoparib) is a novel PARP inhibitor. It has been approved in China as treatment for germline *BRCA1/2*-mutated (g*BRCA1/2*^mut^), platinum-sensitive recurrent OC, and as maintenance therapy for platinum-sensitive recurrent OC, regardless of *BRCA1/2* mutation status [17–19]. In phase 2 and 3 trials, fuzuloparib was well tolerated in OC patients, with a low treatment discontinuation rate due to adverse events (AEs) [17, 18]. Compared with other approved PARP inhibitors, fuzuloparib has a lower risk of gastrointestinal toxicities, which might be related to its postprandial administration and high oral bioavailability [20]. Apatinib is a highly selective VEGFR2-targeted TKI. It has demonstrated efficacy and safety across a wide variety of solid tumors, including OC and BC [21–24]. In a murine xenograft model, the combination of fuzuloparib and apatinib showed enhanced antitumor efficacy compared with apatinib alone, without apparent incremental toxicity [20]. Currently, the oral combination of fuzuloparib and apatinib is undergoing clinical development, aiming to improve treatment convenience and spare patients from the toxicities associated with conventional chemotherapy. In this phase 1 trial, we assessed the safety, clinical activity, and pharmacokinetics (PK) of fuzuloparib plus apatinib in the treatment of patients with advanced OC or TNBC. Furthermore, we conducted biomarker analysis according to g*BRCA1/2* mutation.

## Methods

This multi-center, open-label, dose-escalation and PK-expansion phase 1 study (ClinicalTrials.gov, number NCT03075462) was conducted in 10 centers in China. Patients enrolled were women aged 18–70 years, had confirmed advanced high-grade serous epithelial ovarian, fallopian tube, or primary peritoneal cancer (with or without g*BRCA*^mut^), or TNBC. Patients with OC were required to have received 2–4 prior lines of platinum-based chemotherapy. Both platinum-sensitive (disease progression or relapse ≥ 6 months after the last platinum-based chemotherapy) or resistant (disease progression or relapse within 1–6 months after the last platinum-based therapy) disease were allowed. Patients with TNBC were required to receive ≤ 2 prior lines of chemotherapy for advanced disease, and had disease progression or recurrence during or after the last anti-cancer treatment. Other inclusion criteria were an Eastern Cooperative Oncology Group (ECOG) performance status of 0 or 1, a life expectancy of ≥ 3 months, measurable disease per Response Evaluation Criteria in Solid Tumors (RECIST) v1.1 and adequate organ functions. Key exclusion criteria were prior use of PARP inhibitors or anti-angiogenic agents, central nervous system metastases, uncontrolled hypertension (systolic blood pressure > 150 mmHg or diastolic blood pressure > 90 mmHg), a history of congestive heart failure, bowel obstruction or gastrointestinal bleeding (grade 3 or 4 per Common Terminology Criteria for Adverse Events [CTCAE] v4.03) within 4 weeks.

The trial protocol was approved by the institutional review board or ethnic review committee of each participating center. All procedures were conducted in accordance with the guidelines of Good Clinical Practice and the Declaration of Helsinki. All patients provided written informed consent before enrollment.

### Procedure

The study used a standard 3 + 3 dose escalation design. The starting dose of fuzuloparib was 40 mg twice daily (bid) in combination with a fixed dose of apatinib at 250 mg once daily (qd); fuzuloparib could be dose escalated in 20 mg increments to up to 100 mg. An additional dose level of fuzuloparib 80 mg bid plus apatinib 375 mg qd was planned after assessment of the regimen of fuzuloparib 80 mg bid plus apatinib 250 mg qd. If dose escalation of fuzuloparib to 100 mg was completed, dose escalation for apatinib was initiated with two dose level planned: 375 mg qd and 500 mg qd (with fuzuloparib fixed at 100 mg bid). During dose escalation, the next-higher dose level was opened if less than one-third of patients in the previous dose level experienced a dose-limiting toxicity (DLT). Based on preliminary safety and efficacy data in the dose-escalation cohort, dose levels for PK-expansion were selected, with a total of 8–12 patients enrolled in each level. All patients received a single dose of oral fuzuloparib on day 1 and a single dose of oral apatinib on day 4 in cycle 0 (6 days in total), followed by continuous dosing of fuzuloparib and apatinib in 28-day cycles starting on cycle 1 day 1. Treatment continued until disease progression, intolerable toxicity, or patient withdrawal.

### Outcomes

The primary endpoint was to determine the recommended phase 2 dose (RP2D) and tolerability of the fuzuloparib-apatinib combination. Secondary endpoints included best overall response, objective response rate (ORR) and disease control rate (DCR) per RECIST v1.1, cancer antigen 125 (CA-125) response, PFS, overall survival, and PK parameters.

Safety was monitored using AEs, laboratory tests and clinical examinations. AEs were graded according to CTCAE v4.03. DLTs were assessed from the first dose of study treatment (cycle 0 day 1) to the end of cycle 1 of the combination therapy (34 days in total), and defined as any of the following treatment-related AEs (TRAEs): non-hematologic toxicities of grade 3 or worse (with the exception of well-managed grade 3 nausea, vomiting, or diarrhea, and grade 3 creatinine or electrolyte disorder which resolved to grade 1 or baseline level within 24 h), uncontrolled hypertension of grade 3 or worse, grade 4 neutropenia or grade 3 neutropenia accompanied by fever (≥ 38.5 °C), grade 4 thrombocytopenia and toxic effects resulting in dose delay for ≥ 14 days.

Tumor response was assessed using CT or MRI at baseline and every 2 cycles per RECIST v1.1. A complete response (CR) or partial response (PR) was confirmed with a subsequent scan at least 4 weeks after the initial documentation. Disease evaluations based on CA-125 were performed according to the Gynecologic Cancer Intergroup (GCIG) criteria. CA-125 response, defined as a reduction of ≥ 50% in CA-125 level, was assessed in patients with CA-125 levels ≥ 2 folds of the normal upper limit at baseline; the response was confirmed by repeat testing at least 4 weeks apart.

Pharmacokinetic sampling for single dose of fuzuloparib and apatinib was done on day 1 and day 4 (pre-dose and 0.5, 1, 2, 3, 4, 8, 12, 24, 48 h post-dose) of cycle 0, respectively; sampling for continuous dosing of the combination therapy was done on days 1 (pre-dose and 0.5, 1, 2, 3, 4, 8, 12, 24 h post-dose), 8 (pre-dose), 15 (pre-dose), 22 (pre-dose) of cycle 1, and day 1 (pre-dose and 0.5, 1, 2, 3, 4, 8, 12, 24 h post-dose) of cycle 2.

### Statistical analysis

All enrolled patients who received at least one dose of study drug were included in the efficacy and safety analysis. Patients who received at least one dose of study drug and were evaluable for DLT (experienced a DLT or completed the whole 34-day assessment period without experiencing a DLT) were included in DLT analysis. Treated patients who had post-dose PK data were included in PK analysis. All efficacy analysis was exploratory in nature. Time-to-event endpoints were estimated using the Kaplan–Meier method, with the 95% CIs calculated using the Brookmeyer and Crowley method. PK parameters were calculated using the WinNonlin noncompartmental model. Safety outcomes were summarized using descriptive statistics. All statistical analyses were done using SAS v9.4.

## Results

### Patients

**Fig. 1:**
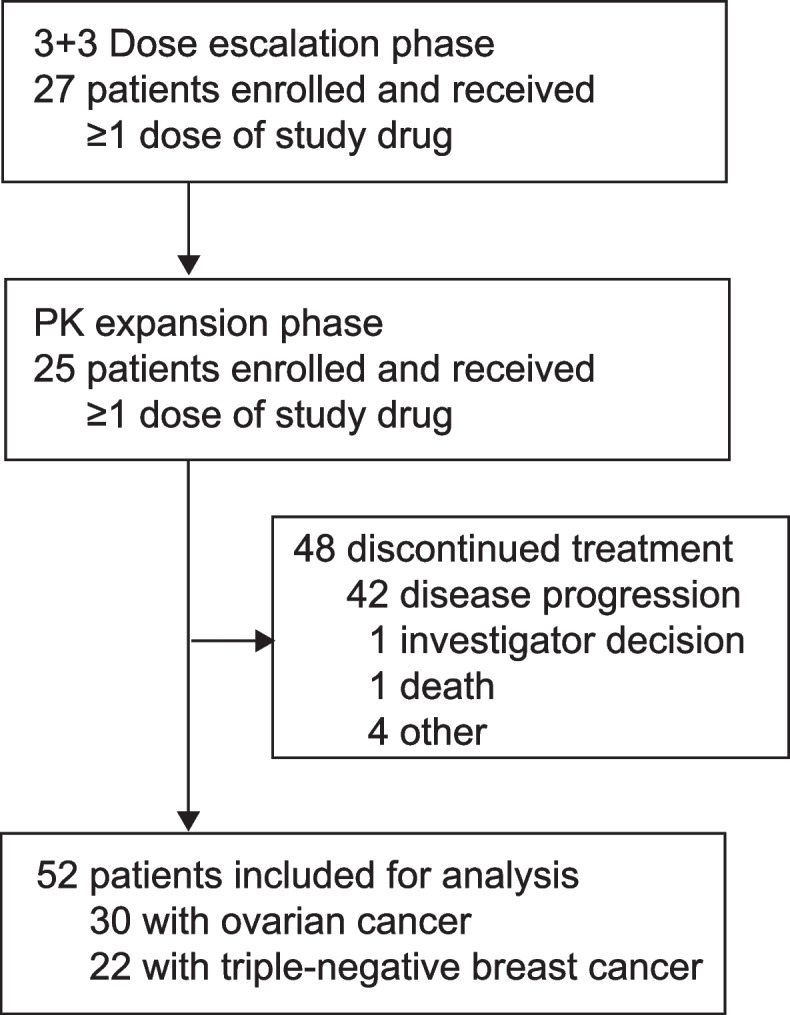
Trial profile

Between Mar 17, 2017 and Mar 2, 2021, 52 patients were enrolled: 27 in the dose-escalation cohort and 25 in the PK-expansion cohort. Of them, 30 patients had OC and 22 had TNBC (Fig. 1). At the data cutoff of Aug. 22, 2021, the median follow-up was 11.3 months (IQR 6.6–20.6). The baseline characteristics are summarized in Table 1. Of the 30 patients with OC, 70% had 2 prior lines of treatment and 30% had more than 2 lines. 40% of patients with OC had invasive metastasis, all with multiple metastatic sites; 26.7% had g*BRCA*^mut^ and 33.3% were platinum-sensitive. Of 22 patients with TNBC, 13.6% had g*BRCA*^mut^.

**Table 1:** Baseline characteristics

	Ovarian cancer (*n* = 30)	Triple-negative breast cancer (*n* = 22)
Age, years	54.5 (44–70)	53 (22–66)
ECOG performance status		
0	19 (63.3%)	18 (81.8%)
1	11 (36.7%)	4 (18.2%)
Histology type		
High-grade serous	30 (100%)	-
No. of lines of previous palliative chemotherapy		
2	21 (70.0%)	22 (100%)
3	7 (23.3%)	0
4	2 (6.7%)	0
Invasive metastasis		
Yes	12 (40.0%)	13 (59.1%)
No	18 (60.0%)	9 (40.9%)
No. of metastasis sites		
1	4 (13.3%)	7 (31.8%)
2	14 (46.7%)	12 (54.6%)
3	6 (20.0%)	3 (13.6%)
≥ 4	6 (20.0%)	0
CA-125		
< ULN	4 (13.3%)	-
≥ ULN- < 2ULN	3 (10.0%)	-
≥ 2ULN	22 (73.3%)	-
*BRCA* mutation status		
Mutation carrier	8 (26.7%)	3 (13.6%)
Wild-type	22 (73.3%)	19 (86.4%)
Platinum status		
Sensitive	10 (33.3%)	-
Resistant	17 (56.7%)	-
Refractory	3 (10.0%)	-

Data are n (%) or median (range). *ECOG *Eastern Cooperative Oncology Group, *ULN *Upper limit of normal

### Dose optimization and safety

During dose escalation, 7 dose combinations, up to fuzuloparib 100 mg bid plus apatinib 500 mg qd, were evaluated. Two of 27 patients in the dose-escalation cohort had DLTs in the first cycle. At the dose level of fuzuloparib 100 mg plus apatinib 250 mg, 1 patient experienced febrile neutropenia and grade 4 decreased white blood cell count; at fuzuloparib 100 mg plus apatinib 375 mg, 1 patient experienced grade 4 decreased platelet count. The maximum tolerated dose (MTD) was not reached.

Overall, 19 (36.5%) of 52 enrolled patients had at least one grade ≥ 3 TRAE, with the most common being hypertension (10 [19.2%] patients), anaemia (7 [13.5%]), and decreased platelet count (3 [5.8%]; Table 2). No patients discontinued any component of study treatment due to TRAE. Eleven (21.2%) patients required a dose reduction or interruption. Serious TRAEs were reported in 6 (11.5%) patients, with the most common being decreased platelet count (2 [3.8%] patients). One death (cerebral hemorrhage/brain herniation) in the fuzuloparib 60 mg plus apatinib 250 mg cohort was considered possibly related to study treatment.

**Table 2:** TRAEs occurring in ≥ 10% of patients

	All patients (*n* = 52)	
	Any grade	Grade ≥ 3
Any TRAE	49 (94.2%)	19 (36.5%)
Hypertension	27 (51.9%)	10 (19.2%)
White blood cell count decreased	20 (38.5%)	2 (3.8%)
Neutrophil count decreased	20 (38.5%)	1 (1.9%)
Nausea	16 (30.8%)	0
Asthenia	14 (26.9%)	0
Anaemia	13 (25.0%)	7 (13.5%)
Dizziness	12 (23.1%)	0
Platelet count decreased	10 (19.2%)	3 (5.8%)
Headache	10 (19.2%)	0
Vomiting	10 (19.2%)	0
Diarrhoea	7 (13.5%)	0
Abdominal pain upper	6 (11.5%)	0
Alanine aminotransferase increased	6 (11.5%)	0
Protein urine present	6 (11.5%)	0

Data are n (%). All grade ≥ 3 TRAEs occurring in ≥ 2% of patients are listed. *TRAE T*reatment-related adverse event

### PK

The PK profiles of fuzuloparib plus apatinib after a single dose and at steady state are shown in Additional file 1: Table S1 and S2 respectively. At the tested dose levels, plasma exposure of fuzuloparib (AUC_0-12 h_) and apatinib (AUC_0-∞_) increased in a dose-dependent manner after a single administration of each agent. After a single co-administration (cycle 1 day 1) of the combination therapy, the plasma exposure of fuzuloparib (AUC_0-12 h_) appeared unaffected by apatinib dosing, whereas the exposure of apatinib (AUC_0-24 h_) was reduced by fuzuloparib dosing (by 15.5%-38.3% across dose levels). After continuous dosing of apatinib in combination with fuzuloparib, the AUC_0-24 h_ of apatinib was reduced by 47.7%-65.0% across dose levels (cycle 2 day 1), compared with the corresponding AUC_0-∞_ after a single dosing of apatinib monotherapy (cycle 0 day 4). The steady-state C_max_ and AUC_0-24 h_ of apatinib increased approximately proportionally with increasing doses (from 250 to 500 mg), when administrated with fuzuloparib at a dose of 100 mg bid.

### Clinical activity

**Fig. 2:**
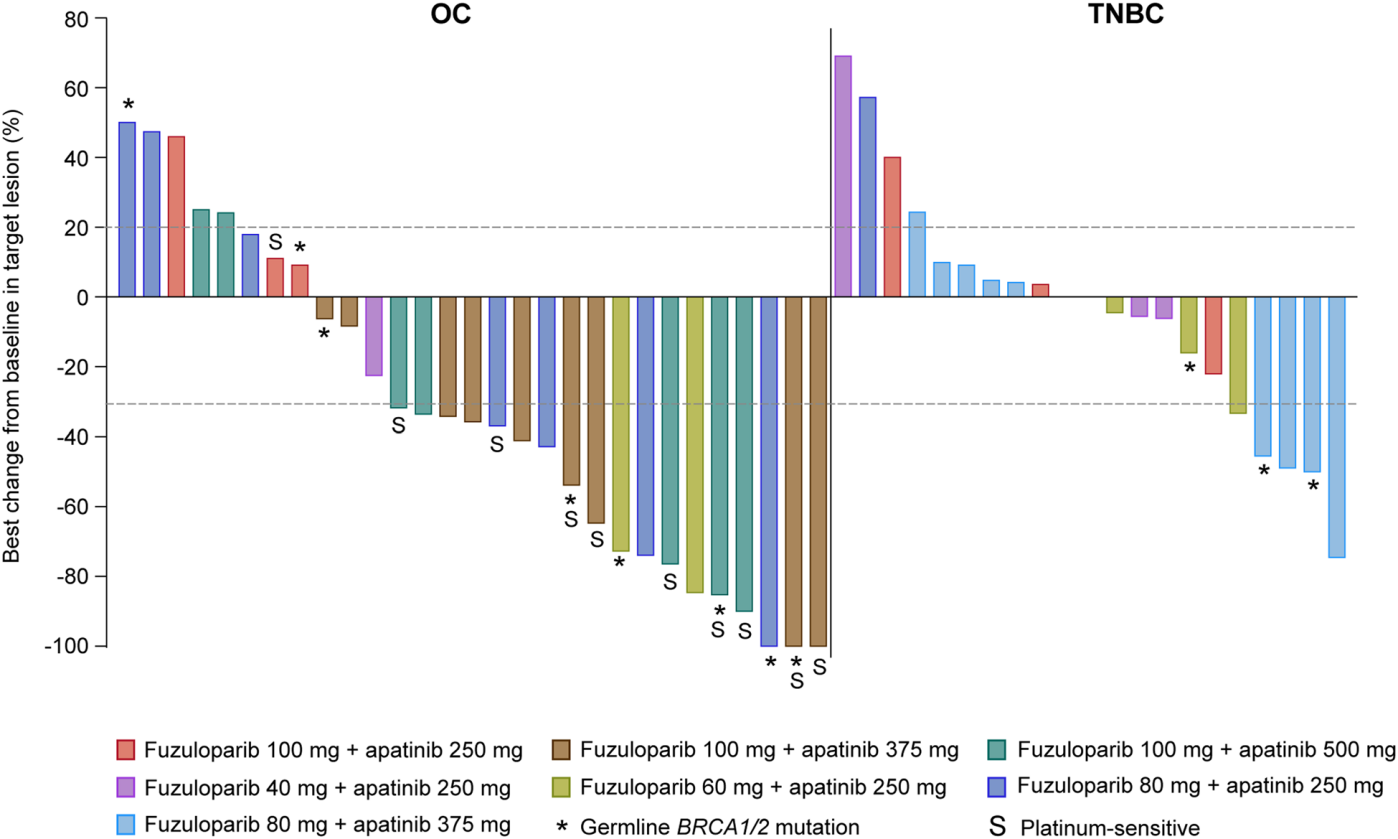
Waterfall plot of the best response in target lesion. Tumor response was assessed by the investigator according to RECIST version 1.1. OC, ovarian cancer; TNBC, triple-negative breast cancer

Change in size of target lesion from baseline for each patient is shown in Fig. 2. Across all dose levels, 5 (9.6%) of 52 patients had CR, 14 (26.9%) had PR, and 15 (28.8%) had stable disease (SD) per RECIST v1.1. The confirmed ORR and DCR in all patients were 36.5% (19/52; 95% CI 23.6–51.0) and 65.4% (34/52; 95% CI 50.9–78.0) respectively. At the highest dose level of fuzuloparib 100 mg plus apatinib 500 mg, the confirmed ORR and DCR were 50.0% (4/8; 95% CI 15.7–84.3) and 62.5% (5/8; 95% CI 24.5–91.5) respectively. Taken together with the general good tolerability and dose-proportional exposure of the combination therapy within tested dose levels, fuzuloparib 100 mg bid plus apatinib 500 mg qd was determined to be the RP2D.

**Fig. 3:**
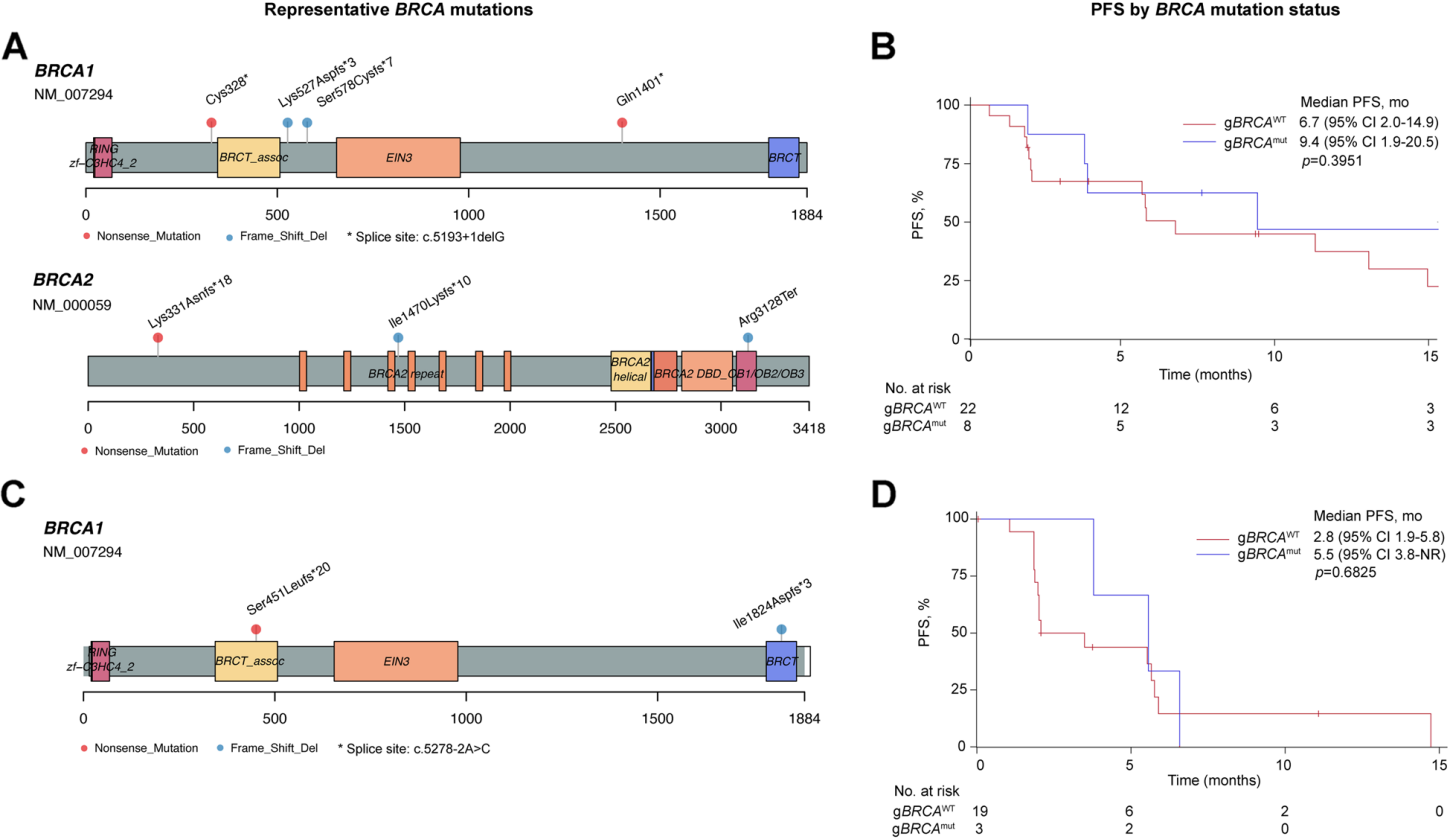
Efficacy by *BRCA* mutation status. Representative *BRCA* mutations and PFS by *BRCA* mutation status in patients with ovarian cancer (**A**, **B**) and TNBC (**C**, **D**). Among 8 patients with g*BRCA*^mut^ ovarian cancer, 5 had g*BRCA1*^mut^, 2 had g*BRCA2*^mut^ and 1 had both g*BRCA1*^mut^ and *gBRCA2*^mut^. Among 3 patients with g*BRCA*^mut^ TNBC, all had g*BRCA1*^mut^. g*BRCA*^mut^, germline *BRCA* mutation; g*BRCA*^wt^*,* wild-type germline *BRCA;* PFS, progression-free survival; TNBC, triple-negative breast cancer

By tumor type, 8 of 10 (80%; 4 CR) patients with platinum-sensitive OC and 6 of 17 (35.3%; 1 CR) patients with platinum-resistant OC achieved confirmed objective response; additional 3 (17.6%) patients with platinum-resistant disease had unconfirmed PR. One (10%) patient with platinum-sensitive OC and 7 (41.2%) patients with platinum-resistant OC had SD, resulting in a DCR of 90.0% (9/10) for platinum-sensitive OC and 76.5% (13/17) for platinum-resistant OC. In responders, median DoR was 9.3 months (95% CI 7.4-not reached [NR]) in patients with platinum-sensitive OC and 18.4 months (95% CI 12.0-NR) in those with platinum-resistant OC (Table 3). By data cutoff, median PFS was 12.1 months (95% CI 1.9-NR) in patients with platinum-sensitive OC and 5.8 months (95% CI 2.1–18.5) in those with platinum-resistant OC. According to *BRCA* mutation status, the proportion of OC patients achieving confirmed objective response (62.5% [5/8] vs 40.9% [9/22]) and disease control (87.5% [7/8] vs 68.2% [15/22]) was numerically higher in those with g*BRCA1/2*^mut^ disease than with g*BRCA1/2* wild type (g*BRCA1/2*^wt^) disease (Additional file 1: Table S3); the median PFS was 9.4 months (95% CI 1.9–20.5) in patients with g*BRCA1/2*^mut^ OC and 6.7 months (95% CI 2.0–14.9) in those with g*BRCA*^wt^ OC (*p* = 0.3951; Fig. 3A, B). In patients who had platinum-sensitive OC, all 3 patients (100%) with g*BRCA1/2*^mut^ and 5 of 7 (71.4%) patients with g*BRCA*^wt^ had an objective response; in patients with platinum-resistant OC, an objective response was observed in 2 of 4 (50.0%) of patients harboring g*BRCA1/2*^mut^ and in 4 of 13 (30.8%) patients harboring g*BRCA*^wt^.

**Table 3:** Efficacy outcomes by tumor type

	Platinum-sensitive OC (*n* = 10)	Platinum-resistant OC (*n* = 17)	TNBC (*n* = 22)	Total (*n* = 52)^a^
Best overall response per RECIST v1.1, n (%)				
CR	4 (40.0%)	1 (5.9%)	0	5 (9.6%)
PR	4 (40.0%)	5 (29.4%)	5 (22.7%)	14 (26.9%)
SD	1 (10.0%)	7 (41.2%)	7 (31.8%)	15 (28.8%)
PD	1 (10.0%)	4 (23.5%)	9 (40.9%)	17 (32.7%)
NE	0	0	1 (4.5%)	1 (1.9%)
Confirmed ORR, % (95% CI)	80.0 (44.4–97.5)	35.3 (14.2–61.7)	22.7 (7.8–45.4)	36.5 (23.6–51.0)
DCR, % (95% CI)	90.0 (55.5–99.7)	76.5 (50.1–93.2)	54.5 (32.2–75.6)	65.4 (50.9–78.0)
Median DoR (95% CI), months	9.3 (7.4-NR)	18.4 (12.0-NR)	4.5 (3.7-NR)	11.1 (7.4–16.6)
CA-125 response, % (95% CI)^b^	100 (47.8–100)	42.9 (17.7–71.1)	-	50.0 (28.2–71.8)
Median PFS (95% CI), months	12.1 (1.9-NR)	5.8 (2.1–18.5)	3.8 (2.0–5.7)	5.7 (3.5–6.7)
Median OS (95% CI), months	NR (20.4-NR)	25.5 (14.9-NR)	10.7 (7.5-NR)	21.1 (14.9–33.9)

^a^Included 3 patients with platinum-refractory OC. ^b^Assessed in 22 patients with OC (5 platinum-sensitive and 17 platinum-resistant) who had CA-125 level above 2 folds of the normal upper limit at baseline. *CR* Complete response, *DCR* Disease control rate, *DoR* Duration of response, *NE* Not evaluable, *NR* Not reached, *OC* Ovarian cancer, *ORR* Objective response rate, *OS* Overall survival, *PFS* Progression-free survival, *PR* Partial response, *PD* Progressive disease, *RECIST* Response evaluation criteria in solid tumors, *SD* Stable disease, *TNBC* Triple-negative breast cancer

In 22 patients with TNBC, 0 CR, 5 (22.7%) confirmed PR and 7 (31.8%) SD were observed and the DCR was 54.5% (12/22; Table 3). In 3 patients harboring g*BRCA*^mut^, 2 (66.7%) PR and 1 (33.3%) SD were recorded. By data cutoff, the median PFS were 5.5 months (95% CI 3.8-NR) in patients with g*BRCA*^mut^ TNBC and 2.8 months (95% CI 1.9–5.8) in those with g*BRCA*^wt^ TNBC (*p* = 0.6825; Fig. 3C, D).

## Discussion

In this phase 1 trial, the chemo-free combination of fuzuloparib and apatinib demonstrated good tolerability and therapeutic efficacy in biomarker-unselected patients with recurrent gynecological cancers. No MTD was established, and no saturation of plasma drug exposure was observed up to fuzuloparib 100 mg bid plus apatinib 500 mg qd.This highest tested dose combination was determined to be the RP2D. The safety profile of the fuzuloparib-apatinib combination was consistent with the individual agents, with no new safety signals identified. The most frequently reported grade 3 or worse TRAEs were hypertension (the most common TRAE associated with apatinib monotherapy [21, 22, 25]) and hematologic toxicities (the most common TRAEs associated with fuzuloparib monotherapy [17–19]). These events were generally manageable with standard supportive care and dose modification, without leading to treatment discontinuation.

In this study, obvious anti-tumor activity was seen for fuzuloparib plus apatinib, with a confirmed ORR of 36.5% and DCR of 65.4% across all dose levels. Specifically, the ORR was 80% for patients with platinum-sensitive recurrent OC, which was much higher than the 35.3% for patients with platinum-resistant recurrent OC. The cross-sensitivity between platinum-based chemotherapy and fuzuloparib-apatinib combination could be partly explained by the overlap between resistance mechanisms of platinum and PARP inhibitor, which involves the reactivation of the HR repair pathway [26]. Notably, the *gBRCA1/2* mutation rate of 26.7% for HGSOC patients in our study was slightly higher than the reported rates of 19–23.8% for *BRCA*-unselected OC patients (> 90% serous histology) in phase 3 trials of other PARP inhibitors [27–29]. This difference could be attributed to the relatively small sample size of this study. Consistent with previous reports [12, 13, 29], *BRCA1/2*^mut^ was predictive of favorable efficacy outcome in OC and the ORR ranged from 30.8% in g*BRCA*^wt^, platinum-resistant disease to 100% in g*BRCA*^mut^, platinum-sensitive disease in this study. In a previous phase 2 trial, fuzuloparib monotherapy achieved an ORR of 69.9% and a median PFS of 12.0 months in patients with g*BRCA*^mut^, platinum-sensitive OC (~ 70% received 2 prior lines of chemotherapy and none received PARP inhibitor) [17]. In this study, our subjects represented a heavily pretreated patient population with recurrent OC, similar to that in the phase 2 trial of fuzuloparib monotherapy. Although the sample size was limited, the ORR and PFS achieved with the combination therapy in patients with platinum-sensitive OC with or without g*BRCA*^mut^, appeared to be comparable to those achieved with fuzuloparib monotherapy in patients with platinum-sensitive OC with g*BRCA*^*mut*^. Given the established superior efficacy of PARP inhibitors in treating patients with g*BRCA*^mut^ OC [13, 29], our preliminary data support the potential clinical benefits with the addition of apatinib.

A handful of clinical trials have assessed the combination of an anti-angiogenic agent and a PARP inhibitor as treatment for recurrent OC, and the ORR ranged from 60%-79.6% in platinum-sensitive disease [11–13, 29] and 11.1%-15.4% in platinum-resistant disease [14–16]. Direct comparison between studies was difficult considering the differences in patients characteristics (e.g. proportion of patients with *BRCA* mutation/HR deficiency) and study design (e.g. no. of lines of prior treatment [chemotherapy, PARP inhibitor, and anti-angiogenic agent] allowed); nevertheless, the ORR of 30.8% with fuzuloparib-apatinib in patients with g*BRCA*^wt^, platinum-resistant OC was encouraging for a difficult-to-treat population. Importantly, tumor response with fuzuloparib plus apatinib in OC was durable regardless of platinum sensitivity or *BRCA* mutation status, which highlights the necessity for developing new biomarker to better identify potential responders in the g*BRCA*^wt^, platinum-resistant OC population. Notably, in a recent phase 3 trial, cediranib plus olaparib failed to significantly extend PFS versus standard chemotherapy (median, 10.4 vs 10.3 months) in patients with platinum-sensitive OC (64.6% with 1 line of prior therapy). This was speculated to be attributed to the decreased dosing intensity (71.6% of participants required dose modification) and increased rate of treatment discontinuation (21.2% withdrew treatment) due to AEs with the combination [29]. In this study, treatment with fuzuloparib plus apatinib resulted in a median PFS of 12.1 months in patients with platinum-sensitive OC progressing after ≥ 2 lines of platinum-based chemotherapy. The combination was well tolerated, with only 21.2% requiring dose modification and none requiring dose discontinuation due to TRAE. The overall favorable safety profile of fuzuloparib-apatinib supports long-term and sustained use of these drugs, which potentially distinguishing this combination from others under development.

Although the PARP inhibitors olaparib and talazoparib have shown robust efficacy in g*BRCA*^mut^ TNBC, the prevalence of this mutation is low, ranging from 10–20% in TNBC [30–32]. A phase 1 trial has evaluated the combination of olaparib with cediranib in pretreated TNBC patients with or without *BRCA1/2* mutation; however, the trial observed limited anti-tumor activity (no objective response and 2 SD in 8 patients), likely due to the small sample size. In the present study, fuzuloparib plus apatinib resulted in an ORR of 22.7% and DoR of 4.5 months in patients with TNBC who had received up to 2 prior lines of chemotherapy. While the clinical activity was modest, the fuzuloparib-apatinib combination as a later-line therapy showed similar effectiveness to conventional chemotherapy and had fewer toxicities in treating a general TNBC population [32, 33], suggesting that it might be considered as chemo-free option under specific conditions. Alternatively, an encouraging ORR of 66.6% and DCR of 100% was obtained with the combination in the subgroup of g*BRCA1/2*^mut^ TNBC. Based on the consistent efficacy of PARP inhibitors shown in *gBRCA1/2*^mut^, HER2- BC, and the promising results observed in phase 1 trials for fuzuloparib with or without apatinib in g*BRCA1/2*^mut^ BC [19], we have initiated a phase 1/3 trial (NCT04296370) to assess fuzuloparib with or without apatinib, compared to investigator's choice of chemotherapy, in the treatment of *gBRCA*^mut^, HER2- BC. This ongoing study includes a run-in phase 1 part, followed by a 3-arm, randomized, phase 3 part. The trial will provide definitive evidence regarding the impact of including apatinib in the treatment regimen for HER2- BC.

When used as combination therapy, the PK profile of fuzuloparib (40–100 mg bid) was similar to that of fuzuloparib monotherapy; however, the exposure of apatinib (250–500 mg qd) at steady state was reduced by 47.7%-65.0% across dose levels when compared to apatinib used as monotherapy. This observation indicates the presence of drug-drug interaction. As the PK profiles of two drugs in multiple doses for combination therapy have been investigated, the data imply that higher dose level of apatinib may be tolerable when combined with fuzuloparib. Besides, the decreased exposure of apatinib suggests potential lower related toxicity of apatinib when used in combination with fuzuloparib. Based on the PK data, it’s recommended that the dose of apatinib be adjusted first in the combination, in the management of intolerable AEs that were potentially related to either agent.

## Conclusions

In summary, the combination of fuzuloparib and apatinib showed hematologic DLTs and acceptable safety in patients with recurrent OC and TNBC. Fuzuloparib 100 mg bid plus apatinib 500 mg qd was determined to be the RP2D. With the activity observed in OC and TNBC, further investigation of the combination therapy is warranted as a potential alternative to cytotoxic treatment.

### Supplementary Information

**Additional file 1:** **Table S1.** Pharmacokinetic parameters of fuzuloparib and apatinib after single dosing. **Table S2.** Pharmacokinetic parameters of fuzuloparib and apatinib after multiple dosing (cycle 2 day 1). **Table S3.** Tumor response in patients with ovarian cancer by germline BRCA mutation status.

## Data Availability

Datasets supporting the conclusions of the study are presented in the paper or are available from the corresponding authors upon reasonable request.

## Abbreviations

AE: Adverse event
bid: Twice daily
CA-125: Cancer antigen 125
CR: Complete response
CTCAE: Common Terminology Criteria for Adverse Events
DCR: Disease control rate
DLT: Dose-limiting toxicity
ECOG: Eastern Cooperative Oncology Group
g*BRCA*: Germline *BRCA*
GCIG: Gynecologic Cancer Intergroup
HGSOC: High-grade serous ovarian cancer
HR: Homologous recombination
MTD: Maximum tolerated dose
ORR: Objective response rate
PARP: Poly (ADP-ribose) polymerase
PFS: Progression-free survival
PK: Pharmacokinetics
PR: Partial response
qd: Once daily
RECIST: Response Evaluation Criteria in Solid Tumors
RP2D: Recommended phase 2 dose
TNBC: Triple-negative breast cancer
TRAE: Treatment-related adverse event
VEGF: Vascular endothelial growth factor
VEGFR: Vascular endothelial growth factor receptor
