# Supplementary material for: A phase 1 trial of fuzuloparib in combination with apatinib for advanced ovarian and triple-negative breast cancer: efficacy, safety, pharmacokinetics and germline BRCA mutation analysis
Source: BMC Med. 2023 Sep 29;21:376. doi: 10.1186/s12916-023-03046-8 (PMC10542255; doi:10.1186/s12916-023-03046-8)
Supplement: Supplementary file 1 — Additional file 1: Table S1. Pharmacokinetic parameters of fuzuloparib and apatinib after single dosing. Table S2. Pharmacokinetic parameters of fuzuloparib and apatinib after multiple dosing (cycle 2 day 1). Table S3. Tumor response in patients with ovarian cancer by germline BRCA mutation status. [file 12916_2023_3046_MOESM1_ESM.pdf]

Supplementary materials

Table S1. Pharmacokinetic parameters of fuzuloparib and apatinib after single dosing.

		FZPL 40 mg + APTN 250 mg	FZPL 60 mg + APTN 250 mg	FZPL 80 mg + APTN 250 mg	FZPL 100 mg + APTN 250 mg	FZPL 80 mg + APTN 375 mg	FZPL 100 mg + APTN 375 mg	FZPL 100 mg + APTN 500 mg
Cycle 0 Day 1 (single dose of FZPL)								
FZPL	T_{max}, h	3.0 (2.0-4.0)	3.0 (2.0-4.0)	3.0 (1.2-4.0)	3.0 (2.0-4.0)	3.0 (1.0-8.0)	2.0 (1.0-8.0)	2.5 (1.0-7.9)
	C_{max}, μg/mL	1.6 (34.7)	1.8 (21.1)	2.7 (20.2)	3.2 (23.2)	2.3 (48.0)	3.5 (37.4)	4.0 (33.7)
	$AUC_{0\text{-}12h}$, h*μg/mL	8.7 (22.7)	12.9 (23.8)	18.8 (23.9)	21.9 (30.7)	16.1 (48.8)	25.3 (37.1)	27.3 (29.1)
Cycle 0 Day 4 (single dose of APTN)								
APTN	T_{max}, h	2.5 (1.9-3.0)	4.0 (2.0-4.0)	3.0 (1.9-3.1)	2.5 (2.0-3.0)	3.0 (2.0-4.0)	3.0 (2.0-4.0)	3.0 (1.9-8.2)
	C_{max}, μg/mL	0.95 (36.5)	0.90 (45.1)	1.0 (28.1)	0.86 (26.9)	0.90 (31.1)	1.2 (40.4)	1.6 (32.6)
	$AUC_{0\text{-}24h}$, h*μg/mL	6.2 (16.2)	6.5 (38.2)	6.3 (21.4)	5.8 (20.0)	7.1 (28.9)	7.7 (19.9)	11.5 (26.8)
	$AUC_{0\text{-}\infty}$, h*μg/mL	7.8 (15.6)	9.6 (41.9)	7.6 (29.1)	7.6 (22.6)	9.4 (30.6)	10.8 (24.8)	17.1 (39.6)
Cycle 1 Day 1 (FZPL + APTN)								
FZPL	T_{max}, h	3.0 (1.0-4.0)	3.0 (3.0-4.0)	3.5 (1.1-8.0)	3.5 (1.0-4.0)	4.0 (2.0-8.0)	3.0 (1.0-8.0)	3.0 (2.0-8.0)
	C_{max}, μg/mL	1.5 (46.0)	1.5 (22.3)	2.8 (27.5)	3.2 (18.1)	2.4 (53.5)	3.5 (28.3)	3.6 (40.6)
	$AUC_{0\text{-}12h}$, h*μg/mL	8.3 (11.3)	11.9 (24.6)	19.7 (21.0)	22.4 (21.0)	16.9 (52.0)	24.9 (31.3)	26.8 (42.0)
APTN	T_{max}, h	2.5 (2.0-3.0)	3.0 (1.0-3.9)	3.0 (2.0-4.0)	2.5 (1.1-4.0)	3.0 (2.0-8.0)	2.0 (1.0-4.0)	3.0 (1.0-4.0)
	C_{max}, μg/mL	0.86 (64.6)	0.68 (35.7)	0.83 (41.7)	0.70 (54.0)	0.79 (37.5)	1.1 (48.4)	1.3 (40.2)
	$AUC_{0\text{-}24h}$, h*μg/mL	4.3 (27.4)	4.5 (35.4)	4.0 (35.2)	3.6 (29.8)	5.2 (25.1)	6.4 (26.9)	9.1 (33.9)

Data are median (range) or geometric mean (geometric coefficient of variation%). APTN, apatinib; $AUC_{0\text{-}12h}$, area under the concentration-time curve from time 0 to 12 h; $AUC_{0\text{-}\infty}$, area under the concentration-time curve from time 0 to infinity; $AUC_{0\text{-}24h}$, area under the concentration-time curve from time 0 to 24 h; C_{max}, peak plasma concentration; FZPL, fuzuloparib; T_{max}, time to reach C_{max}.

Table S2. Pharmacokinetic parameters of fuzuloparib and apatinib after multiple dosing (cycle 2 day 1).

	FZPL 40 mg + APTN 250 mg	FZPL 60 mg + APTN 250 mg	FZPL 80 mg + APTN 250 mg	FZPL 100 mg + APTN 250 mg	FZPL 80 mg + APTN 375 mg	FZPL 100 mg + APTN 375 mg	FZPL 100 mg + APTN 500 mg
FZPL							
T_{max}, h	2.0 (1.0-3.0)	3.0 (1.1-4.0)	2.5 (1.9-4.0)	4.0 (2.0-8.0)	3.0 (2.1-4.0)	3.0 (1.3-4.0)	3.0 (1.0-3.0)
C_{max}, μg/mL	3.2 (5.5)	3.9 (36.0)	4.9 (16.5)	5.5 (22.3)	4.9 (21.6)	6.2 (40.0)	6.5 (30.4)
AUC_{0-12h}, h*μg/mL	22.8 (12.3)	31.1 (33.9)	40.7 (17.9)	48.2 (22.2)	40.8 (19.9)	48.7 (35.6)	54.3 (35.1)
C_{min}, μg/mL	1.0 (39.9)	1.7 (42.5)	2.2 (33.2)	2.8 (30.7)	2.3 (24.6)	2.4 (54.9)	2.9 (50.7)
C_{avg}, μg/mL	1.9 (12.2)	2.6 (33.9)	3.4 (18.1)	4.0 (22.4)	3.4 (19.6)	4.1 (35.8)	4.6 (35.2)
Cl_{ss}/F, L/h	1.8 (12.3)	1.9 (33.9)	2.0 (17.9)	2.1 (22.2)	2.0 (19.9)	2.1 (35.6)	1.8 (35.1)
DF, %	109.0 (26.0)	80.8 (49.9)	76.8 (27.9)	65.2 (38.6)	75.6 (20.7)	83.2 (64.8)	76.3 (26.5)
R_{ac} (C_{max})	2.1 (45.5)	2.6 (22.1)	1.8 (25.3)	1.7 (35.7)	2.4 (29.5)	1.8 (35.0)	2.0 (46.8)
R_{ac} (AUC)	2.7 (20.9)	2.6 (18.7)	2.1 (21.9)	2.2 (25.6)	2.8 (21.9)	2.0 (26.1)	2.2 (45.4)
APTN							
T_{max}, h	1.5 (1.0-4.0)	3.0 (1.1-4.0)	2.0 (0.5-2.1)	2.0 (2.0-4.0)	1.5 (0.0-3.0)	2.0 (1.3-3.1)	3.0 (1.0-4.0)
C_{max}, μg/mL	0.71 (65.7)	0.61 (68.3)	0.76 (57.6)	0.54 (32.2)	0.69 (64.5)	0.91 (23.3)	1.2 (41.3)
AUC_{0-24h}, h*μg/mL	4.1 (58.5)	3.7 (33.3)	3.4 (41.2)	2.7 (18.2)	4.3 (57.2)	4.5 (24.6)	7.0 (28.8)
C_{min}, μg/mL	0.025 (94.4)	0.027 (85.6)	0.019 (58.4)	0.019 (28.2)	0.040 (78.6)	0.039 (49.1)	0.052 (63.6)
C_{avg}, μg/mL	0.17 (58.4)	0.15 (33.3)	0.14 (41.3)	0.11 (18.2)	0.18 (57.1)	0.19 (24.8)	0.29 (29.1)
Cl_{ss}/F, L/h	61.4 (58.5)	68.1 (33.3)	74.3 (41.2)	91.8 (18.2)	87.6 (57.2)	82.6 (24.6)	71.5 (28.8)
DF, %	404 (16.5)	376 (38.5)	525 (17.8)	458 (25.9)	358 (57.2)	455 (18.3)	400 (33.8)
R_{ac} (C_{max})	0.83 (55.5)	0.90 (45.4)	0.92 (46.2)	0.73 (74.4)	0.88 (86.9)	0.92 (63.2)	0.94 (35.9)
R_{ac} (AUC)	0.98 (44.7)	0.82 (30.8)	0.79 (26.7)	0.74 (32.5)	0.85 (73.4)	0.68 (43.8)	0.75 (30.9)

Data are median (range) or geometric mean (geometric coefficient of variation%). APTN, apatinib; AUC_{0-12h}, area under the concentration-time curve from time 0 to 12 h; AUC_{0-24h}, area under the concentration-time curve from time 0 to 24 h; C_{avg}, average plasma concentration; Cl_{ss}/F, apparent clearance at steady state; C_{max}, peak plasma concentration; C_{min}, minimum plasma concentration; DF, degree of fluctuation; FZPL, fuzuloparib; T_{max}, time to reach C_{max}; $R_{ac,}$ accumulation ratio.

Table S3. Tumor response in patients with ovarian cancer by germline *BRCA* mutation status.

	Platinum-sensitive OC		Platinum-resistant OC		All OC*	
	Germline *BRCA1/2*^{mut}	Germline *BRCA1/2*^{wt}	Germline *BRCA1/2*^{mut}	Germline *BRCA1/2*^{wt}	Germline *BRCA1/2*^{mut}	Germline *BRCA1/2*^{wt}
No. of patients	n=3	n=7	n=4	n=13	n=8	n=22
Best overall response per RECIST v1.1, n (%)						
Complete response	1 (33.3%)	3 (42.9%)	1 (25.0%)	0	2 (25.0%)	3 (13.6%)
Partial response	2 (66.7%)	2 (28.6%)	1 (25.0%)	4 (30.8%)	3 (37.5%)	6 (27.3%)
Stable disease	0	1 (14.3%)	2 (50.0%)	5 (38.5%)	2 (25.0%)	6 (27.3%)
Progressive disease	0	1 (14.3%)	0	4 (30.8%)	1 (12.5%)	7 (31.8%)
Not evaluable	0	0	0	0	0	0
Confirmed ORR, % (95% CI)	100 (29.2-100)	71.4 (29.0-96.3)	50.0 (6.8-93.2)	30.8 (9.1-61.4)	62.5 (24.5-91.5)	40.9 (20.7-63.6)
DCR, % (95% CI)	100 (29.2-100)	85.7 (42.1-99.6)	100 (39.8-100)	69.2 (38.6-90.9)	87.5 (47.3-99.7)	68.2 (45.1-86.1)

* Included 3 patients with platinum-refractory OC. DCR, disease control rate; NR, not reached; OC, ovarian cancer; ORR, objective response rate.
